# Supplementary figures and images for: Foveolar Drusen Decrease Fixation Stability in Pre-Symptomatic AMD
Source: Invest Ophthalmol Vis Sci. 2024 Jul 8;65(8):13. doi: 10.1167/iovs.65.8.13 (PMC11232898; doi:10.1167/iovs.65.8.13)

# Power spectra accross groups - vertical motion

## Drifts only

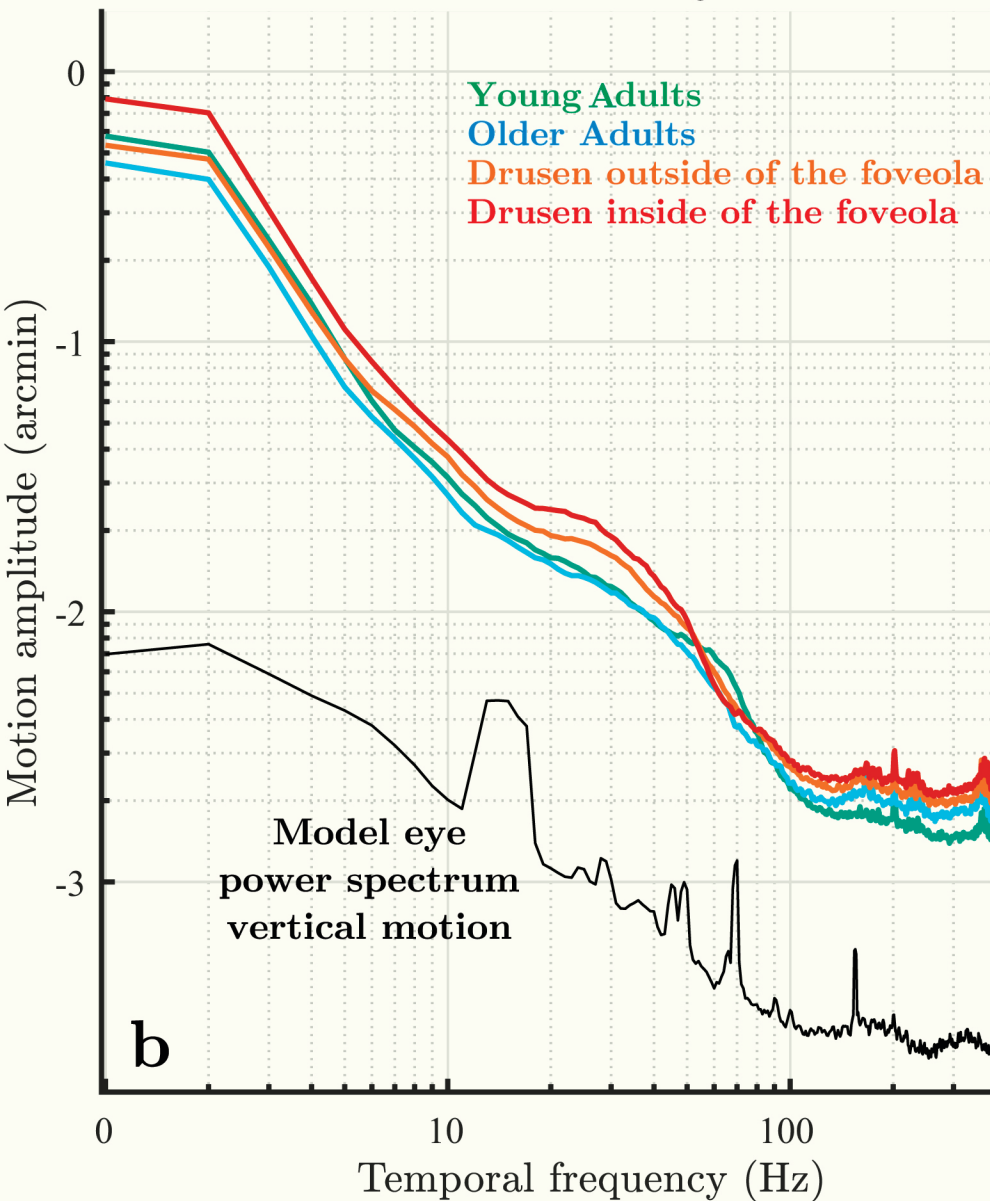

## With microsaccades

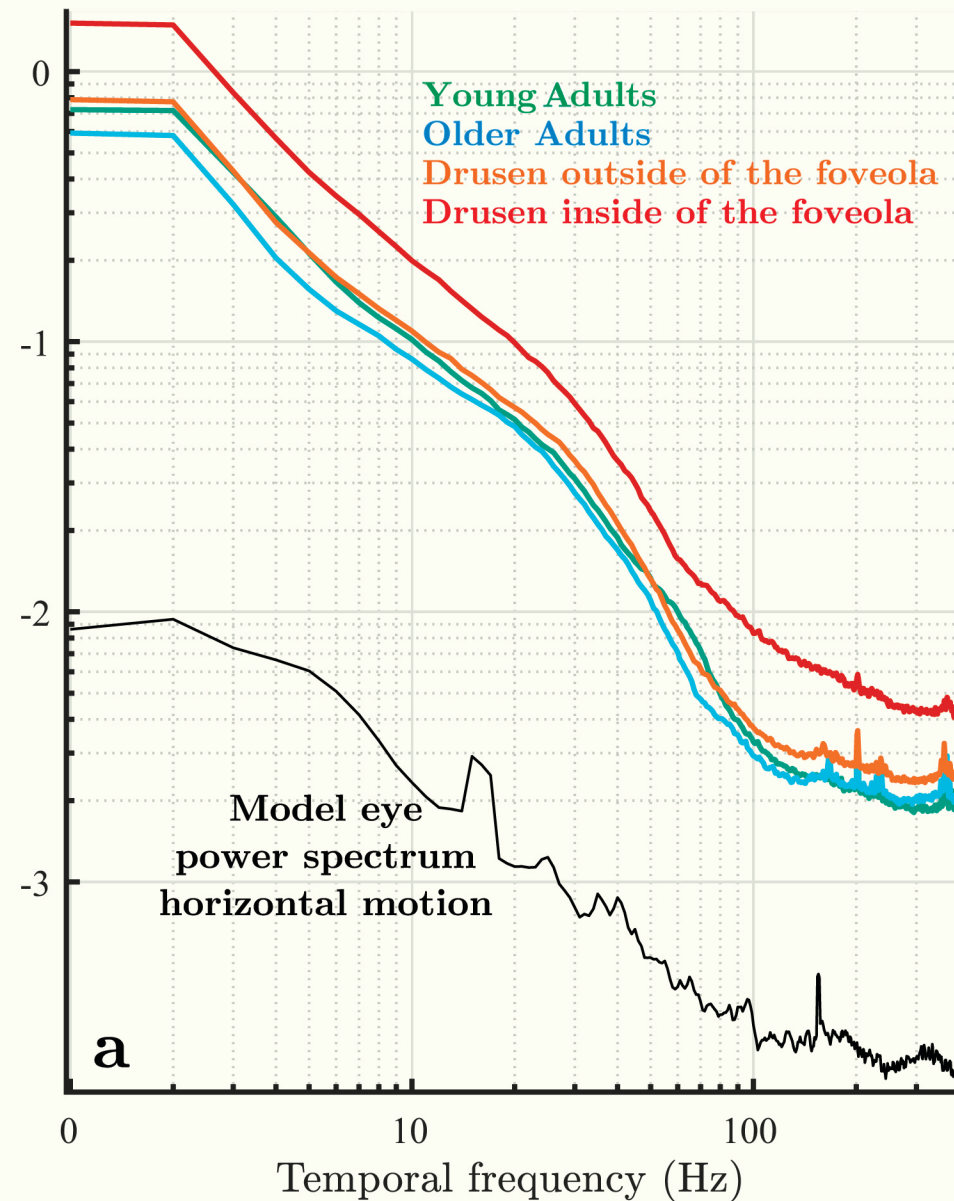

Supplement: Supplement 1 [file iovs-65-8-13_s001.pdf]

0.5°

Young  
Control

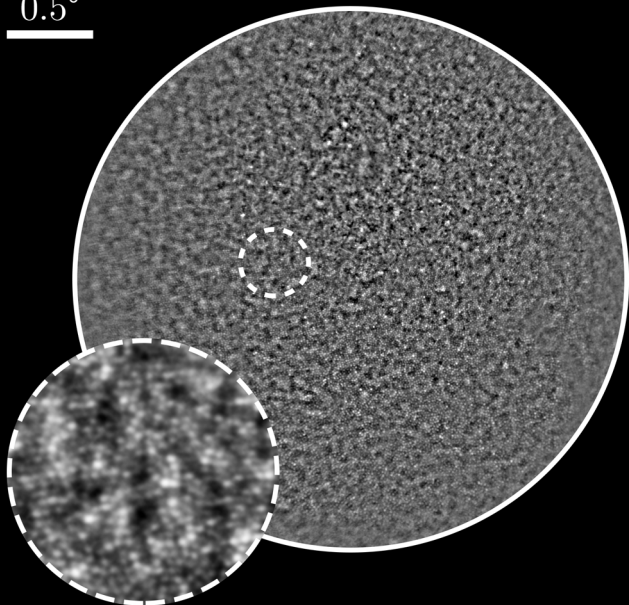

Older  
Control

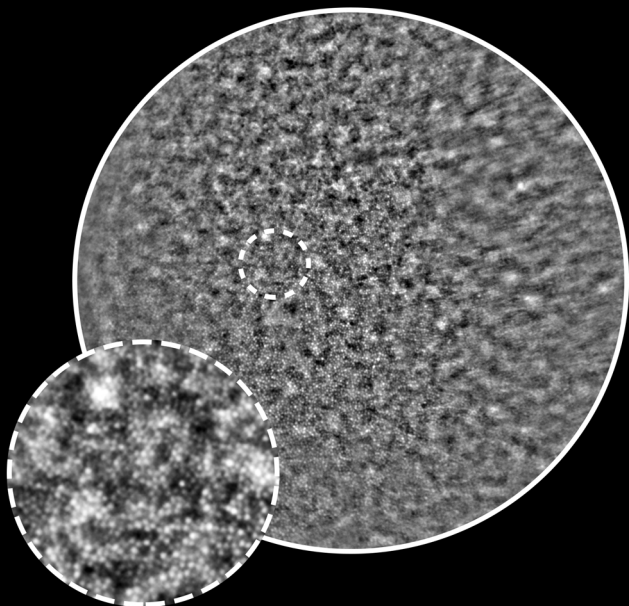

Participants with  
foveal drusen  
and eye opacity

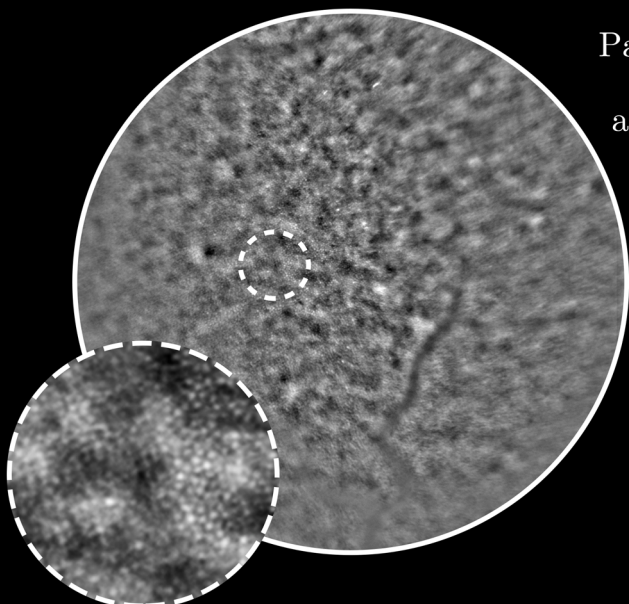

Supplement: Supplement 2 [file iovs-65-8-13_s002.pdf]
