# Supplementary material for: Foveolar Drusen Decrease Fixation Stability in Pre-Symptomatic AMD
Source: Invest Ophthalmol Vis Sci. 2024 Jul 8;65(8):13. doi: 10.1167/iovs.65.8.13 (PMC11232898; doi:10.1167/iovs.65.8.13)
Supplement: Supplement 3 [file iovs-65-8-13_s003.pdf]

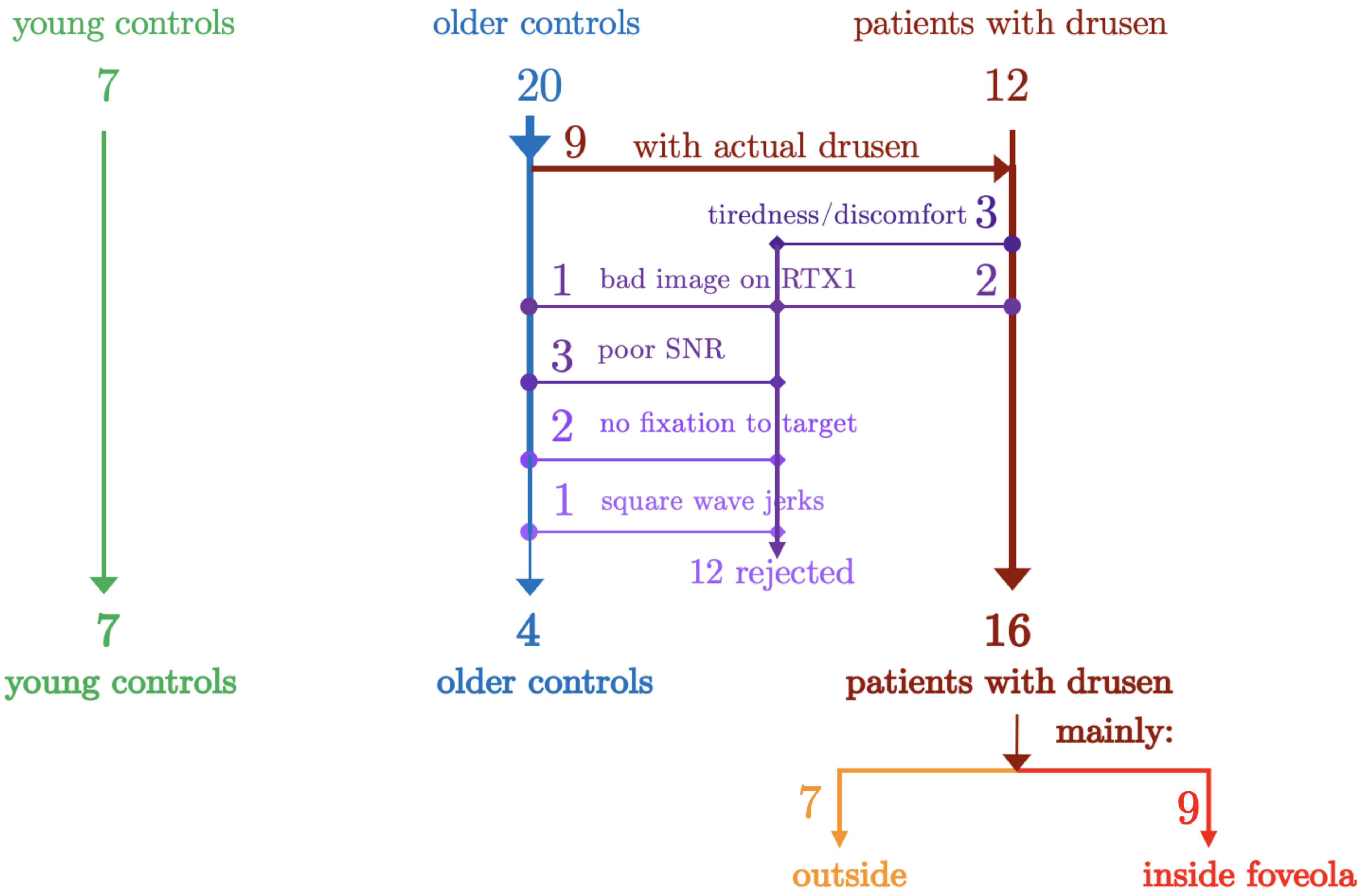
young controls
older controls
patients with drusen
7
20
12
9
with actual drusen
tiredness/discomfort 3
1
bad image on RTX1
2
3
poor SNR
2
no fixation to target
1
square wave jerks
12 rejected
7
4
16
young controls
older controls
patients with drusen
mainly:
7
9
outside
inside foveola
